# Himalayan glaciers experienced significant mass loss during later phases of little ice age

**DOI:** 10.1038/s41598-017-09212-2

**Published:** 2017-09-04

**Authors:** Mayank Shekhar, Anshuman Bhardwaj, Shaktiman Singh, Parminder S. Ranhotra, Amalava Bhattacharyya, Ashish K. Pal, Ipsita Roy, F. Javier Martín-Torres, María-Paz Zorzano

**Affiliations:** 1 Birbal Sahni Institute of Palaeosciences, Lucknow, India; 2 Division of Space Technology, Department of Computer Science, Electrical and Space Engineering, Luleå University of Technology, Luleå, Sweden; 3 Institut für Kartographie, Technische Universität Dresden, Dresden, Germany; 4 Department of Environmental Science, Sharda University, Greater Noida, India; 5 Instituto Andaluz de Ciencias de la Tierra (CSIC-UGR), Armilla, Granada, Spain; 6 Centro de Astrobiología (INTA-CSIC), 28850 Torrejón de Ardoz, Madrid, Spain

## Abstract

To date, there is a gap in the data about the state and mass balance of glaciers in the climate-sensitive subtropical regions during the Little Ice Age (LIA). Here, based on an unprecedented tree-ring sampling coverage, we present the longest reconstructed mass balance record for the Western Himalayan glaciers, dating to 1615. Our results confirm that the later phase of LIA was substantially briefer and weaker in the Himalaya than in the Arctic and subarctic regions. Furthermore, analysis of the time-series of the mass-balance against other time-series shows clear evidence of the existence of (i) a significant glacial decay and a significantly weaker magnitude of glaciation during the latter half of the LIA; (ii) a weak regional mass balance dependence on either the El Niño-Southern Oscillation (ENSO) or the Total Solar Irradiance (TSI) taken in isolation, but a considerable combined influence of both of them during the LIA; and (iii) in addition to anthropogenic climate change, the strong effect from the increased yearly concurrence of extremely high TSI with El Niño over the past five decades, resulting in severe glacial mass loss. The generated mass balance time-series can serve as a source of reliable reconstructed data to the scientific community.

## Introduction

The youngest and highest Himalayan mountains sustain enormous fresh water reservoirs in the form of snow, glaciers, natural lakes, permafrost and wetlands, and support a population of ~1.3 billion^1^. In fact, the Hindu Kush-Himalaya (HKH) harbors ~50% (by area)^1^ of all the glaciers outside of the polar regions which are also vulnerable to minute changes in temperature and precipitation regimes due to their latitudinal positions^1^. The uncertainties in the understanding of the status of glaciers and climate change in the Himalayan region are high due to the scarce and fragmented glacio-meteorological records, owing to the difficult terrain and inclement weather conditions^2, 3^. The increased frequency of extreme weather events and glacial disasters in these high mountains warrants a glaciological database and chronology to better assess the past and present glacier-climate relationship, and the future hydrometeorological scenarios^1, 4^. The assessment of the retreating Himalayan glaciers^5^ has generated considerable concern regarding the changing climate and its widespread effects on the future natural resources^6, 7^.

The difference between snow/ice accumulation and loss, i.e., the mass balance of a glacier, is the most consistent and direct indicator of its health and response to the changing climate^1, 8^. However, existing glacier mass balance records display poor temporal and spatial coverage with a discreet and non-uniform global distribution that signifies the even poorer availability of glacial records for the climate-sensitive subtropical mountains such as the Himalaya^1^. The absence of precise historical or dendroglaciological records is the reason why we have the so little knowledge about the state of the Himalayan glaciers during the Little Ice Age (LIA)^9^. For the tropical and sub-tropical regions, the Little Ice Age is usually marked between 1500 CE and 1850 CE^10–12^. There are already several disagreements on the concept^9, 13^, driving factors^14, 15^, duration^16^, prevalence^17, 18^, synchronicity^19–21^, and climatological characteristics^22^ of the LIA. We certainly cannot generalize the LIA response of the Arctic and subarctic glaciers to the lower latitude Himalayan ones as glaciers can display dissimilar behavior even in similar regional climatic settings due to varying geometry and bed topography^9^. Reaching a global understanding of the LIA effects, at different latitudes and altitudes, is essential to comprehend the past and future implications of drastic transient climate fluctuations. In addition to investigating dendroglaciology at the local scale, the detection of regional scale climate signals also need proper exploration since the glacier mass balance shows high cross-correlations across regional scales^8^. However, in the Himalayan context, a reconstructed regional mass balance record based on tree-ring data or any other proxy is unavailable mainly due to the hardships of reliable field data collection in the difficult terrain. There were several exploratory attempts to investigate the relationship of tree-ring width with Himalayan hydroclimatology^23–28^ and recent glacier movements^29–31^.

Our research is the maiden attempt to reconstruct the longest regional scale glacier mass balance records for the Western Himalaya based on tree-ring sampling at an unprecedented scale. Another highlight of our study is that it presents valid evidence of the significant mass loss experienced by the Himalayan glaciers even during the LIA. A comparative analysis with the Arctic and subarctic glaciers reveals that the Himalayan glaciers behaved differently during the LIA, thus indicating that the LIA was substantially briefer and weaker in the Himalayan region. We demonstrate an improved understanding of the temporal-regional glacier-climate relationship with significant implications for El Niño and Total Solar Irradiance (TSI). The generated mass balance time-series can serve as a source of reliable reconstructed data to the scientific community.

## Sampling and LIA trends

**Figure 1:**
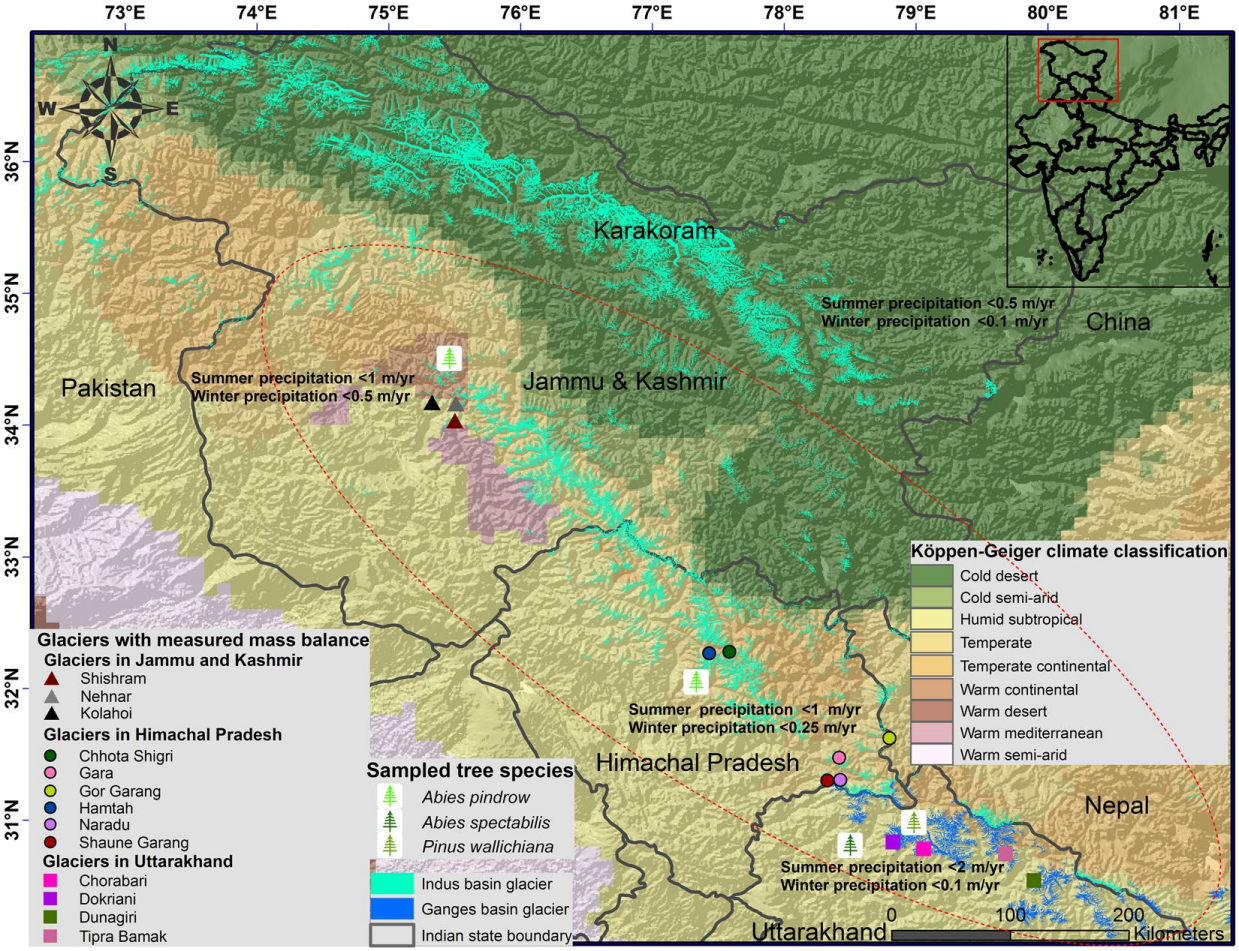
Study area. The study provides a representative mass balance time-series for ~10,000 glaciers within similar climate zones in the Western Himalaya (within red dotted ellipse). The data used to generate the broad climate categories were obtained from the supplementary material from Peel *et al*.^32^ and the information about the precipitation regimes was inferred from Burbank *et al*.^33^. The glacier outlines are taken from http://rds.icimod.org/Home/Data?group=15&&themekey=HKH&&page=1&&themekey=HKH. The background hillshade image was generated using a 90 m spatial resolution Shuttle Radar Topography Mission (SRTM) Digital Elevation Model (DEM) (http://rds.icimod.org/Home/DataDetail?metadataId = 8744). The inset map displays the location of the study area marked by the red rectangle. The map is created using ArcGIS Version 10.4 (http://desktop.arcgis.com/en/arcmap/latest/get-started/setup/arcgis-desktop-quick-start-guide.htm).

In this study, we have selected the study area based on the availability of all the glaciological mass balance measurements and the corresponding tree-ring sampling in the Indian Himalaya (Fig. 1). The study provides a representative mass balance time-series for ~10,000 glaciers within similar climate zones in the Western Himalaya within the red dotted ellipse in Fig. 1. A short introduction to the dendroglaciology is given in the materials and methods section, as is a detailed account of the data collection, statistical analysis, mass balance reconstructions, and validations. We have performed separate data collection and analysis for the glaciers in the three Indian Himalayan states which represent different climates and precipitation regimes and significantly contribute to the Indus and Ganges river systems (Fig. 1). The northernmost state of Jammu and Kashmir (J&K) displays the highest climatic variability, with a mixture of temperate continental, Mediterranean, temperate and humid subtropical towards the west and south-west and cold arid and semi-arid towards east and north-east^32^. The J&K glaciers under consideration here fall within the temperate continental climate zone and mostly gain ice mass through winter snowfall caused by the Western Disturbance (WD)^33^. This study does not include the Karakoram glaciers in eastern and north-eastern J&K because they represent completely different topographical and climatic controls and lack high altitude tree-line and measured glaciological mass balances. The Himalayan state south of J&K is Himachal Pradesh (HP) and the HP glaciers fall within the temperate and temperate continental climate zones^32^. These glaciers receive snowfall from both the WD in the winter and the Indian Summer Monsoon (ISM). The J&K and HP glaciers contribute to the Indus river system while the glaciers in the southernmost Himalayan state of Uttarakhand (UK) contribute to the Ganges river system. The UK glaciers fall mainly within the humid subtropical climate and receive snow mainly from ISM. Thus, although all of the Himalayan glaciers receive varied WD contributions to their mass budget^34, 35^, the J&K glaciers represent the monsoon shadow zone, the HP glaciers signify the monsoon transition zone, and the UK glaciers are monsoon dominated; accordingly, these western Himalayan glaciers warrant separate considerations for their mass balance reconstructions.

**Figure 2:**
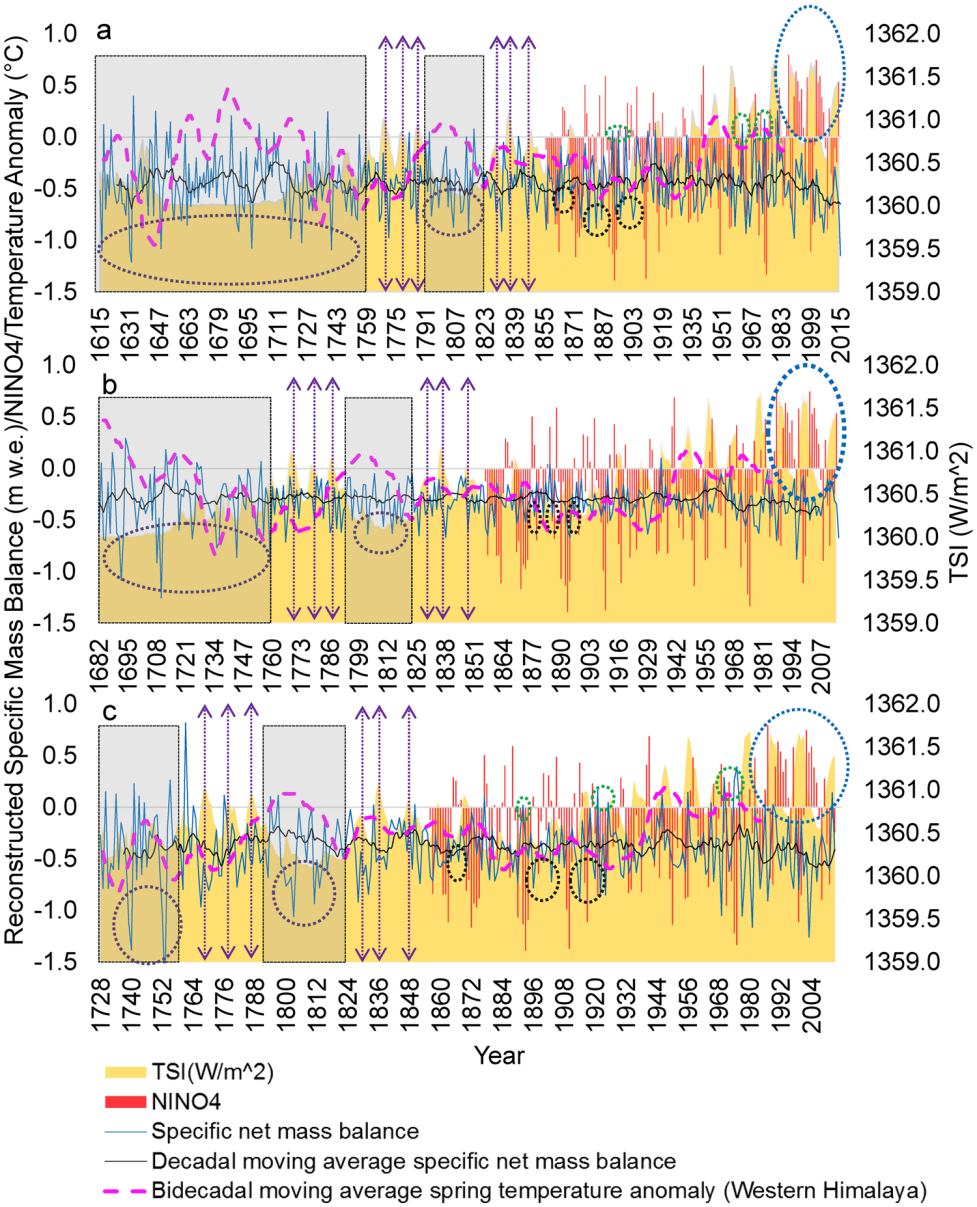
Reconstructed specific mass balance for the Himalayan glaciers. (**a**) UK glaciers. (**b**) J&K glaciers. (**c**) HP glaciers. Gray shaded regions highlight the years with positive mass balance and the lowest TSI during the LIA. Violet ellipses within the gray shading highlight a few of the most negative mass balances during the lower solar activity in the LIA, probably due to an enhanced El Niño affecting ISM, as is evident in the latter years with high Niño 4 index (black ellipses). Purple arrows signify the direct relationship between high TSI and more negative mass balances during the LIA. Green ellipses show positive mass balances in the post LIA years with low TSI and negative Niño 4 index. Blue ellipses highlight the increased coincidences of El Niño with extremely high TSI since the 1970s. The reconstructed mean spring temperature departure time-series is taken from Yadav and Singh^37^, the TSI time-series is obtained from http://spot.colorado.edu/~koppg/TSI/TSI_TIM_Reconstruction.txt, and the Niño 4 index is derived from http://www.esrl.noaa.gov/psd/gcos_wgsp/Timeseries/Nino4/ ref. 38.

**Figure 3:**
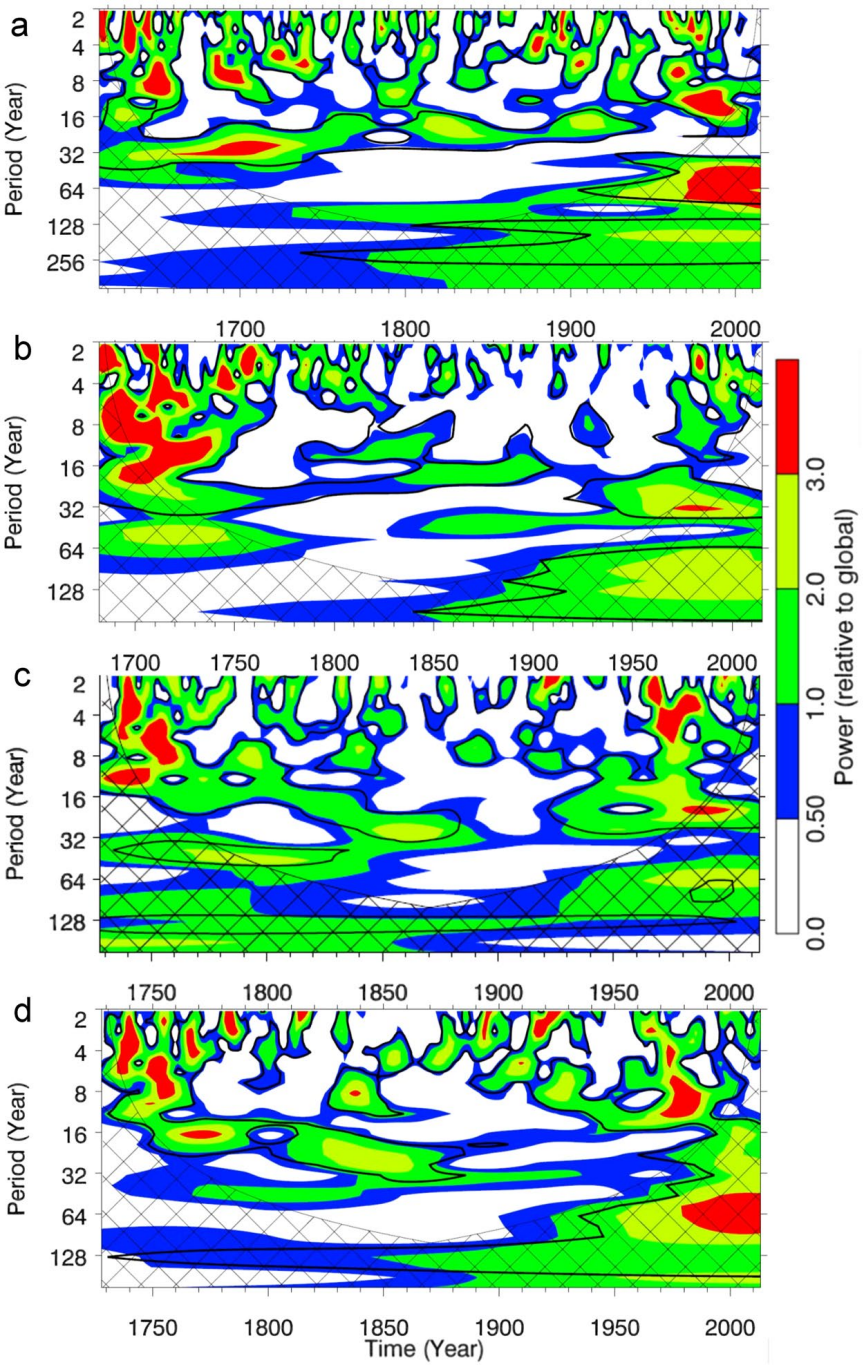
Wavelet power spectrum. (**a**) UK glaciers. (**b**) J&K glaciers. (**c**) HP glaciers. (**d**) Western Himalaya. Plots are prepared using an online tool (http://ion.exelisvis.com)^40^. The black contours are the 10% significance regions above the global wavelet spectrum (GWS). Netted region represents the cone of influence.

We present the regional mass balance reconstruction results in Fig. 2. The longest reconstructed time-series goes back to 1615 CE for the UK glaciers (Fig. 2a), followed by 1682 CE for J&K (Fig. 2b), and 1728 CE for the HP glaciers (Fig. 2c). Our first objective is to visualize the glacier mass balance scenarios during the latter half of the LIA which is marked by a considerable decrease in the solar activity particularly during 1645 CE – 1715 CE (Maunder minimum) and 1790 CE – 1830 CE (Dalton minimum)^36^. The gray shaded regions in Fig. 2 highlight the years with the highest positive mass balance and lowest Total Solar Irradiance (TSI) during the LIA. However, the violet ellipses within the gray shading in Fig. 2 also mark the years with extremely negative mass balances even during low solar activity in the LIA. These observations strikingly coincide with the long-term tree-ring-based spring temperature patterns in the western Himalaya^37^ (pink dotted curve in Fig. 2). The extremely negative mass balances in the gray shaded regions can easily be seen flanking the positive temperature anomaly years. If we go ahead in this time-series (~1880 CE – 1940 CE) in Fig. 2, we find several more years highlighted by black ellipses when although the TSI was lower, the mass balances were significantly negative due to the positive Niño 4 indices. This highlights a significant dependence of the UK (Fig. 2a) and southern HP (Fig. 2c) glaciers not only on the temperature conditions but also on the ISM precipitation. The reverse of this effect can be observed for the years marked by the green ellipses in Fig. 2, which show positive mass balances in the post LIA years with low TSI and negative Niño 4 indices. At the same time, the J&K glaciers (Fig. 2b), which are at the highest altitudes and the highest Himalayan latitudes are relatively far from industrial settlements and dense populations, and receive significant winter snowfall from the WD, show the least fluctuation in their mass balance profile due to their lack of dependence on the ISM and regional temperature instabilities. Thus, we believe that the episodes of significantly negative mass balances (violet ellipses) during the gray shaded phases in the LIA (Fig. 2) were the result of an enhanced El Niño affecting the ISM and increasing the temperatures, while the purple arrows (Fig. 2) further signify a more direct relationship between the high TSI and more negative mass balances during the LIA in the years with potentially weaker El Niño. These visual observations and inferences are well-supported by the results of the wavelet analysis (Fig. 3) described below. Wavelet decomposition studies are especially relevant to the analysis of non-stationary systems, i.e., systems with short-lived transient components, such as those observed in climate-related series, and the wavelet analysis permits the analysis of the relationships between two different signals.

Figure 3 shows the results of an advanced time–frequency analysis that we performed by carefully considering the recommendations (detailed in the materials and methods section) given by the pioneer studies^39, 40^ describing wavelet transformations for climate time-series. The periodicities and oscillations of the regional mass balances derived from the robust wavelet analysis display a spectacular congruence with the observations made in Fig. 2. For the UK glaciers, the lowest reconstructed mass balance corresponds to 1631 CE (Fig. 2a), during the LIA. It follows a positive temperature anomaly in the preceding years, and the equivalent time-frequency graph in Fig. 3a suggests a periodicity of ~2–5 years corresponding to strong El Niño episodes in those years^41^. Similarly, the next significantly low mass balance in 1648 CE (Fig. 2a), can be attributed to another short but strong El Niño episode with a periodicity ~2–4 years (Fig. 3a). However, flanking these El Niño episodes, another significant periodicity of ~8–10 years during ~1640–1655 CE is noticeable (Fig. 3a) and can be attributed to the North Atlantic Oscillation (NAO) variability. The dropping temperatures and multiple positive mass balances during this period (Fig. 2a) are thus understandable as being related to the positive phase of the NAO and the warm phase of the El Niño-Southern Oscillation (ENSO) intensifying the WD and bringing precipitation to the Himalaya^42^. Interestingly, the entire period from ~1660–1720 CE has an ~32 year periodicity (Fig. 3a), a possible mark of the Atlantic Multi-decadal Oscillation (AMO)^42–44^, coupled with an NAO episode of ~8 year periodicity in between from 1685-1700 CE (Fig. 3a). This is the reason behind the absence of any ENSO periodicity during this interval, as the warm phase of the AMO is known to break the ENSO-NAO relationship and simultaneously results in higher than average temperatures (Fig. 2a) over the western Himalaya^42^. The NAO episode in the middle of this AMO periodicity can be responsible for the considerable spring precipitation in the Himalaya^42^ and thus for the positive mass balance during ~1685–1690 CE (Fig. 2a,b). In case of the J&K glaciers, the blank spaces in most of the time-series (Fig. 3b) indicate that, as mentioned above, due to their latitudinal and altitudinal setting, they receive significant winter snowfall from the WD and thus show the least fluctuation in their mass balance profile due to their lack of dependence on the ISM and regional temperature fluctuations. Their dependence on the strong WD events is the reason why they show the strongest power bands during the end of 17^th^ and the start of 18^th^ centuries (Fig. 3b). A significant part of this power spectrum during the early 18^th^ century falls within the cone of influence and is not as well resolved as for rest of the Himalaya (Fig. 3b). The time-series for the HP glaciers are the shortest, with a significant span within the cone of influence (Fig. 3c). However, they also show some periodicities of ~2–8 years around ~1730–1760 CE as did the UK and J&K glaciers (Fig. 3c), with several occurrences of positive mass balances (Fig. 2c), thus indicating a prolonged response to the strong AMO episode at ~1660–1720 CE. Figure 3d displays an average picture of the glacier mass balance in the time-frequency domain for the entire western Himalaya. For a larger part of ~1800–1950 CE, any significant periodicity in the reconstructions is absent, as this is the period when the deforestation, population explosion, and industrialisation in these regions started at unprecedented scales. This fact, combined with weaker and sparser El Niño episodes and a significant cold phase of the AMO during ~1880–1930 CE^42^, led to widely mixed and aperiodic responses of the Western Himalayan glaciers to the climate during this period. Nevertheless, in the past 5 decades, several significant trends have been observed which are detailed in the next section.

## Comparisons and recent trends

**Figure 4:**
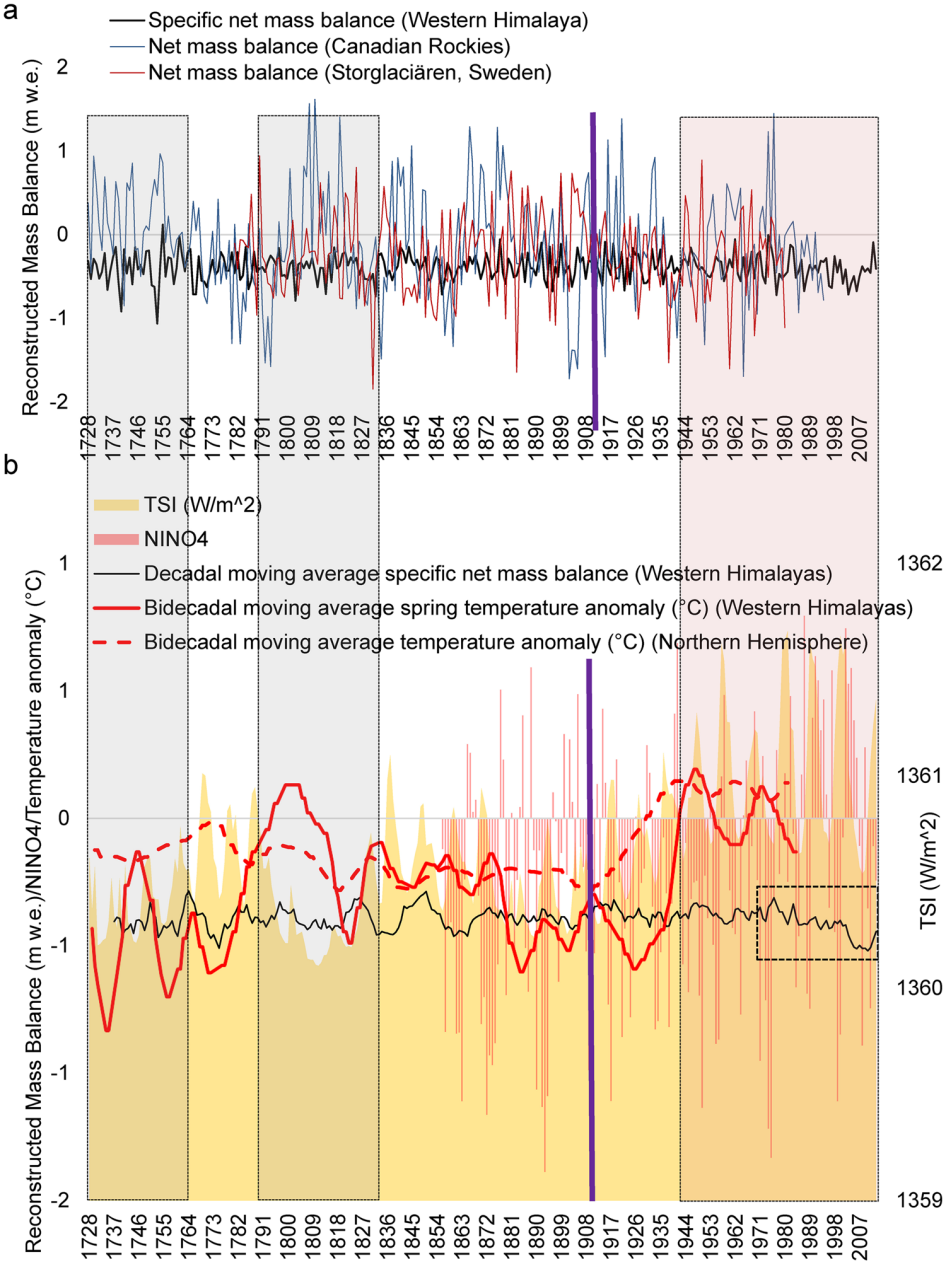
Mass balance comparisons between the Arctic, subarctic, and Himalayan glaciers. (**a**) Yearly mass balance time-series. (**b**) Decadal moving average mass balance time-series. The dashed black rectangle shows the recent period (1970–2010) of decreasing mass balance trend. The gray shaded regions correspond to the years with the lowest TSI during the LIA. The red shaded region highlights the start of strong solar cycles that in later years (since the 1970s) started showing substantial coupling with strong El Niño episodes and rising regional temperatures. The violet line represents the time when the Northern Hemisphere temperatures started experiencing a steep rise due to the cumulative effects of industrialisation. The mass balance data for Sweden are taken from Linderholm *et al*.^45^, and those of the Canadian Rockies are from Lewis and Smith^46^. The Northern Hemisphere temperatures are derived from Mann *et al*.^20^ and the mean spring temperature departure time-series for the western Himalaya is taken from Yadav and Singh^37^.

In Fig. 4, we show the variable responses of the Arctic, subarctic, and Himalayan glaciers to the LIA and post-LIA climate conditions. One thing to consider is that the mass balance for the Arctic^45^ and subarctic glaciers^46^ as shown in Fig. 4 is the net yearly mass balance while that of the Himalayan glaciers is the specific net yearly mass balance. Thus, although the magnitudes of the mass balances seem highly variable and incomparable, our main focus in this discussion will be on the positive or negative values and the trends of the mass balances. The gray shaded regions in Fig. 4 correspond to the years of lowest TSI during the LIA, and throughout these phases, we observe the highest consistency in the positive mass balances for the Arctic and subarctic glaciers (Fig. 4a). As already highlighted in the previous section and visible in Fig. 4, the Himalayan glaciers during these phases did not show many positive mass balances due to a combination of vital climatic scenarios which decide the regional glacial regime. In fact, our observations, given in Figs 2 and 4, are quite consistent with a recent study^47^ that compiled multiproxy records to infer that the glacial advance in the Himalaya occurred prominently up to 1600 CE during the LIA unlike the Canadian, European, and Icelandic glaciers which showed significant advances throughout the LIA. This study^47^ also establishes that the advancement of the glaciers during late-Holocene and LIA was significantly widespread at least in the Central Himalaya, i.e., for the UK glaciers^47^. A speleothem, which is another proxy for reconstructing past climate, collected from a cave at an elevation of ~1520 m in western Uttar Pradesh (south of the location of UK glaciers) showed cooler and wetter conditions during ~1450–1889 CE^48^ along with the confirmed presence of a warmer and drier climate flanking this period due to weak South Asian monsoon^48^. However, Rowan^47^ has clearly compiled several instances of glacier advances during this phase of the LIA from the region, thus confirming the importance of precipitation in addition to the lowered temperatures for the advances of glaciers in the Himalayan Ranges. Several studies^48–50^ have further confirmed the seasonal control of westerly winter precipitation in the southern latitudes of UK glaciers, which happens to be even greater in case of the HP and J&K glaciers. On the similar lines, the complex nature of precipitation (monsoon/westerly)-temperature regimes in these mountains have additionally been long-established^51^ by the fact that the recorded LIA glacier advances are negatively correlated with the hemispheric temperature records and thus, significantly driven by the patterns of westerlies^47, 51^. Due to the westerly influence, the LIA in the Western Himalaya was slightly delayed than the eastern part^47^. However, another study^52^ based on lacustrine sediments suggests the start of cold conditions since 1300 CE, lasting throughout the LIA. We also observed similar scenarios where the UK glaciers are the ones showing the largest mass balance variability, followed by the HP glaciers, and finally by the most consistent profile for the J&K glaciers (Fig. 2).

**Figure 5:**
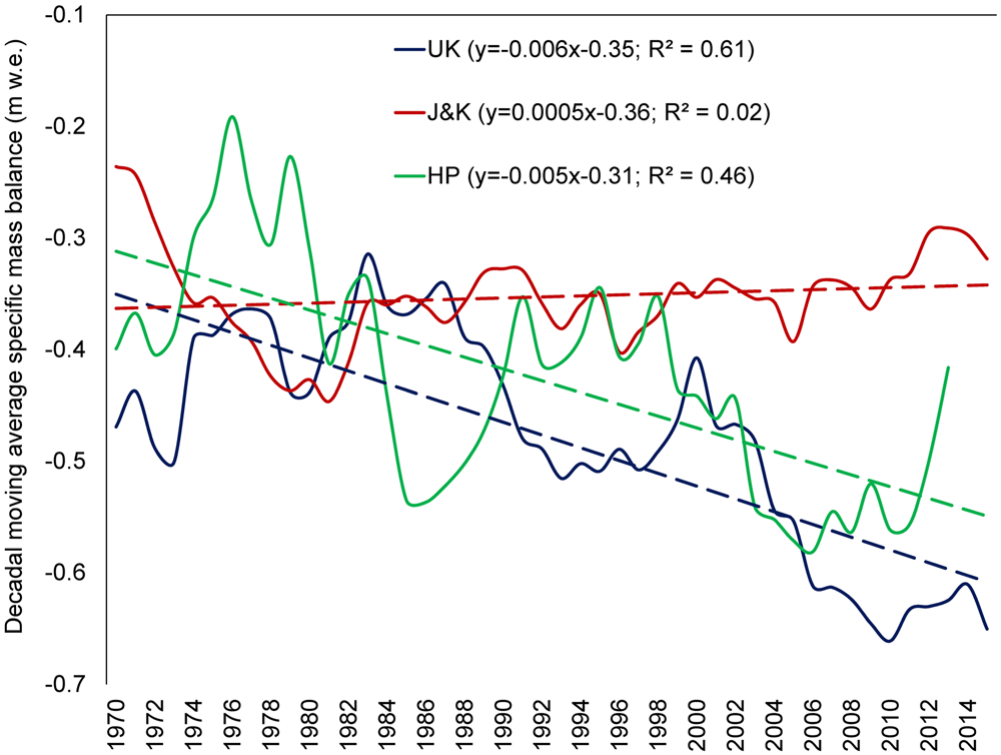
Mass balance trends since 1970 for glaciers in all three regions.

The differences in the LIA responses of these Northern Hemisphere glaciers from across varying latitudes are easily noticeable (Fig. 4). However, the main aim of this section is to highlight the more subtle responses post-LIA, particularly since the start of the second decade of the 20^th^ century. The violet line in Fig. 4 shows the period when the Northern Hemisphere temperatures^20^ started experiencing a steep unidirectional rise. During the 1930s, the Northern Hemisphere temperature anomaly became positive, and, from this point onwards, the number of positive mass balance years in Arctic Sweden and subarctic Canada showed a significant decline (Fig. 4a). In the case of the Himalaya, the red shaded region in Fig. 4 highlights this phase of rising regional temperatures, and the start of the strong solar cycles that in later years (Since the 1970s), started showing substantial coupling with strong El Niño episodes. Figure 3a and d also shows mass balance periodicities of 9–12 years during ~1970–1990, a representation of the response to a few of the strongest consecutive solar cycles in past 400 years^53^. In fact, we see that ~50% of the years since 1970 experienced an exceptionally high TSI of >1361 Wm^−2^, ~40% of which also underwent warm phases of ENSO. These decades are also the ones which have seen unprecedented increase in regional populations, industrialisation, emissions, and deforestation. This makes the distinction between the different contributions from natural and anthropogenic drivers difficult to determine, as Fig. 4 (dashed black rectangle) shows the significantly decreasing mass balance per year since 1970 for the Himalayan glaciers. In fact, only 6 years in the past 5 decades have shown positive regional scale mass balances (Fig. 2). This significant trend of decreasing mass balance has never been so persistent for the western Himalaya (Fig. 4b). The current solar cycle and the subsequent ones are expected to be particularly weak and the mass balance response of the Himalayan glaciers in the coming decades can help us to clearly resolve the degrees of contribution from the natural and anthropogenic drivers. However, there is one more way to achieve this resolution up to a certain extent, as is shown in Fig. 5, i.e., by considering regional mass balance deterioration. While the decadal rates of decrease in mass balance for the UK and HP glaciers (Fig. 5) are ~6% and ~5%, respectively, with a significant yearly correlation in their declines, the decadal rate of decrease for the J&K glaciers (which are the farthest from anthropogenic influences^54^) is nearly stable (Fig. 5). The reason behind the moderate trend of mass loss in J&K can be attributed to the increasing precipitation^55^ and the lower rate of warming^56^ in the region. Although, the J&K glaciers have shown a moderate mass loss trend throughout their time-series, the recent trends for all three Himalayan subregions suggest that the anthropogenic drivers are certainly accentuating the glacier mass loss in the regions which are lower in latitude and altitude and are closer to the population.

## Conclusions and future prospects

The present study aims to provide the first reconstruction of the longest Himalayan glacier mass balance time-series since 1615 CE. These data can be invaluable to the research community to make hydroclimatic inferences about the glaciated Himalayan terrain during the past 400 years. The results also establish that the temporal extent of the LIA was considerably shorter in terms of glacier advances in the Himalaya and that the Himalayan glaciers suffered significant mass losses even during the latter half of the globally accepted age of the LIA. This study further displays the complex climatic controls on the Himalayan glacier mass balances which make them behave in a considerably different manner than the glaciers in higher latitudes. Our results suggest that the Western Himalayan deglaciation started much earlier in the LIA. The tree-ring based spring-to-summer (March–April–May) temperature reconstruction of the UK Himalaya^57^ suggests that this region was relatively warmer during the LIA and did not indicate any centennial cold excursions. This indicates that the LIA was identified as a minor event at least in terms of the changes in spring temperatures, and that the period 1600–1700 CE was generally warm during springs^57^. Further, the tree samples collected from Lahaul-Spiti (HP)^57^ indicate that this region was also warm even during the period 1400–1500 CE, followed by a decrease in the summer temperatures to the coldest during 1700–1900 CE. Thus, we see that even between the adjoining states of UK and HP, the temperatures displayed significant spatio-temporal variability. This variability is even more pronounced if we compare the Eastern Himalaya with the western part. Krusic *et al*.^58^ reconstructed summer temperature variability over the Bhutanese Himalaya showing three prominent cold periods. Two of these periods were in the mid-fifteenth century, and the third one occurred in the late seventeenth century, followed by the warmest period in the first decade of the twenty first century coinciding with the timing of general glacier recession in the Eastern Himalaya^58^. The LIA in the Western Himalaya was not as conspicuous a phenomenon as in the higher latitudes of the Northern Hemisphere. Instead, in the Western Himalaya, LIA was indeed a disconnected phenomenon where periods of warmer episodes intruded the periods of cooler episodes. Our results also highlight the fact that glaciation and deglaciation are resultants of not only temperatures but also precipitation and this complex control of precipitation and temperature regimes is extremely prominent in the Himalaya. Our mass balance time-series suggests that even during several advances in the LIA^47^, there have been phases when significant deglaciation occurred within the Himalaya and the advances showed by some glaciers should not be generalised for the entire mountain range.

Although, the study acknowledges the contributions of anthropogenic drivers of climate change in the Himalayan region, it also highlights a strong effect from the increased yearly concurrence of extremely high TSI with El Niño in the past five decades, resulting in severe glacial mass loss. A global study by Solomina *et al*. (2016)^59^ also correlates the glacier advances with the increased volcanism and low solar irradiance and deglaciations with the anthropogenic activities, thus depicting the significance of external controlling factors on the glacier mass balances. While the instrumental records are inadequate to convincingly assess the extent of anthropogenic climate change, ~180 years of industrial-era warming has already resulted in rising surface temperatures above the preindustrial values, and natural background variability for several regions including the subtropical latitudes in which the Himalaya is situated^60^. Although, this external anthropogenic forcing can partly control the glacial regime in the Himalaya, the natural climate variability still emerges as the key deciding element governing the Himalayan glacier mass balances^5^. Similar to several other studies for the region^30, 43, 54^, our study also identifies ENSO, NAO, and AMO as the primary drivers of the regional mass balance variability. The fact that the past few decades have experienced intensified episodes of NAO, closely correlating with rising temperature^61, 62^ also suggests a robust natural climatic control over the Himalayan glaciers.

Glacier mass balance is a function of a combination of various climatic factors, and we never expected a direct correlation of our reconstructed mass balance time-series with any particular parameter over an extended temporal scale. Such individual controls can occur during their extremes with short spatio-temporal domains, and we have identified several such controls throughout the time-series using visual comparative analysis and wavelet transforms. In fact, the results that we obtained strongly point towards the diverse regional climatic scenarios, complex topographical controls, and an increasing population pressure on the Himalayan glaciers. These are also the main factors that govern the tree-ring growth in the highest tree-lines closest to the glaciers and this is the reason behind our successful glacier mass balance time-series reconstruction, which not only shows a fairly acceptable performance (explained in the validation section of the materials and methods section) but also correlates with the findings of other significant paleoclimatic studies for the region^26, 37, 42, 47^.

This study is a significant step in the direction of establishing glacial chronologies in the Himalayan region. The scope of our research is not to study the advances but to specifically focus on the deglaciation and our results show that the deglaciation in Himalaya in general, and in Western Himalaya in particular, started much before the global average start of deglaciation following the LIA. We agree that the time period between 1500 to 1850 CE denoted as the LIA^10–12^ is debatable in the Indian context. Our study covers major part of the LIA time span and for the initial LIA stages, we are in continuous effort to collect suitable dendrochronological records. We understand that there is still a need for considerable future work, in terms of expanding the sample sites across the Himalaya by including the Eastern Himalaya and Brahmaputra river basin glaciers. This proposed sampling must consist of both, the dendrochronology (tree-ring) samples from the proximity of the glaciers, and the observed glacier mass balances across the Himalayan mountain ranges at longer and consistent spatio-temporal scales. Another improvement for model development and the resolution of the uncertainties would account for the complex topographical differences by sampling multiple adjacent glaciers with different terrain characteristics.

## Materials and Methods

To reconstruct the glacier mass balance records for past few centuries, several botanical proxies based on lichenometry^63, 64^, wood density^65^ and tree-ring width^64, 66^ have been used. However, the lichenometry results lack a necessity for reconstructing annual mass balances, which is an annual resolution^67^. Tree-ring chronologies from trees with strong climate signals are capable of providing this annual resolution^66^. The years with high winter snow accumulation and moderate summers favor positive glacier mass balance while providing unfavorable growing conditions for trees, thus resulting in narrow annual tree-rings, and vice-versa^68, 69^. This inverse relationship between the meteorological parameters controlling the glacier mass balance and the tree-ring width of climatically sensitive species provides a basis for reconstructing the mass balance in remote and inaccessible glacial environments. The first results of the relationship between tree-ring variability, meteorological parameters, and glacier ice volume were reported in the 1960s^70^ followed by subsequent developments^31, 44, 68, 69, 71–76^. Below we describe the methodological steps taken in order to obtain the results detailed in this paper.

### Tree-ring data

Tree rings of many of the conifers growing in the diversified geographical regions of the Himalaya are found datable and suitable for the analysis of the changing climate^72, 77^. We collected tree-ring samples (increment cores) from conifers growing at the upper limits of the subalpine forests close to the snouts of the glaciers from three locations in the Central and Western Himalayan regions (Supplementary Fig. 1). The samples were taken from *Abies spectabilis* (from two sites 78 tree/112 cores and 28 tree/35 core, respectively) and *Pinus wallichiana* (83 tree/122 cores). We included an additional site from the North-West Himalaya (Fig. 1) for the tree-ring width data of *Abies pindrow* (9 tree/10 cores). These data were taken from the NOAA website (http://www.ncdc.noaa.gov/paleo/treering.html) and samples were collected from Sonmarg, Kashmir close to the Thazwas or Tajwah Glacier. These data were updated later by replication of the tree-ring width data of the same species collected in 2015 from the same region with an increased sample size of 14 tree/27cores. Each of these sampling sites is representative of a particular climate regime (Fig. 1). We selected the trees growing at the uppermost tree line zone due to their proximity to the glaciers. The katabatic winds blowing through the glaciers significantly regulate the growth of these nearby trees, thus providing sensitive tree-ring sequences comparable to the changing Equilibrium Line Altitudes (ELA) of the glacier, which responds to climatic variations. We dated the tree-ring sequence of these cores using visual matching of growth patterns using the “skeleton plot” method of cross-dating^78^. We measured the dated tree-ring widths using the LINTAB measuring system and TSAP-Win software^79^. The quality of the cross-dating of the tree-ring width series was further checked using the COFECHA program^80^. Tree-ring width data series were detrended using cubic smoothing splines with a 50% frequency response cutoff of 30 years to make the ring width indices using the ARSTAN program^81^. This also eliminated age-related growth trends and maximised the signal from common factors influencing tree growths. All of the tree-ring width indices were combined to form a tree-ring chronology. We used the residual tree chronology of the Central, Western and North western Himalayan regions to minimize the effect of autocorrelation (Supplementary Fig. 2). The suitability of these chronologies was assessed on the basis of their statistical parameters namely, mean sensitivity (MS), the average correlation between all series (Rbar), the expressed population signal (EPS), and the first-order autocorrelation of the series. The Rbar and EPS values were computed using a 30-year moving window with a 15-year overlap (Supplementary Fig. 3).

### Tree-ring chronologies

The relation between mass balance and tree growth is a function of several climatic drivers. Trees growing closer to the snout are more sensitive to the local environment created by the nearby glaciers. A study of katabatic winds and their patterns in Rongbuk Valley^82^, north of Mount Everest suggests that these wind play a great role in changing the local circulation patterns. The katabatic winds are extremely strong, regular, and prominent in the high Himalaya. The reason for this is that even though the glaciated area in high mountains is not as large as the ice sheets, the larger high altitude glaciers which control the glacio-hydrology of the region prominently are capable of generating such wind patterns daily in the afternoon and the narrow valleys in these mountains further channelise and intensify the flow of these winds downslopes. Based on this premise of significant glacier control over nearby tree growth, we generated tree-ring index chronologies from four sites with three conifers, namely, *Abies pindrow* (Thazwas Glacier-J&K), *Abies pindrow* (Solang Valley-HP), *Abies spectablis* (Dokriani valley-UK), and *Pinus wallichiana* (Gangotri valley-UK) to reconstruct the mass balance. The tree-ring chronologies from the Dokriani valley and the Gangotri valley were found to be positively correlated to each other (r = 0.6, p < 0.001) and were merged to make a regional chronology representative for the entire UK. The residual ring-width index chronologies of these taxa along with the number of samples (cores) included in these chronologies is shown in Supplementary Fig. 2. The mean sensitivities for these chronologies are 0.26, 0.29, 0.18, and 0.17 with the mean correlations (Rbar) of 0.21, 0.17, 0.18, and 0.29 and the signal-to-noise ratios of 5.44, 6.50, 16.20, and 30.23 for *Abies pindrow* (Thazwas Glacier), *Abies pindrow* (Solang Valley), *Abies spectablis* (Dokriani valley), and *Pinus wallichiana* (Gangotri valley), respectively (Supplementary Table 1). The mean sensitivity values of these chronologies indicate that they have high inter-annual variability and display strong environmental signals.

### Mass balance and climate data

We reconstructed the regional mass balance of the Central, Western and NW Himalaya on the basis of the correlations between the tree-ring width indices and the available glaciological mass balance data for the corresponding geographical regions. Supplementary Table 2 shows the details of the observed mass balance data of the glaciers in the region used for reconstruction and the period for which the mass balance data is available. The measured glaciological balance for the Himalayan glaciers is extremely scarce and here we have used all the available and published glaciological mass balance records in order to produce the best possible time-series.

Climate is the common factor influencing both mass balance and tree growth. We analysed the tree growth-climate connection (Supplementary Fig. 4) to validate the mass balance-tree-ring width relationship (Supplementary Fig. 5). This analysis also proved the sensitivities of the sampled tree species to the high altitude Himalayan climate. Observational temperature and precipitation data in the Himalayan region is scarce and fragmented^1^. In addition, the weather stations are located at distant locations from the sampling sites. Therefore, we have used a gridded dataset^35^ covering the sampling locations to ensure the better reliability of the correlation analysis. The temperature data from the National Center for Environmental Prediction and the National Center for Atmospheric Research (NCEP/NCAR)^83^ for 1948–2017 and the precipitation data from the Global Precipitation Climatology Project^84^ for 1901–2013 was used to analyze the correlation with tree-ring width indices (Supplementary Figure 4). We used the average monthly temperatures and the total monthly precipitation starting from October of preceding year to September of the current growth year for all the sites to correlate with the tree-ring indices.

The correlation analysis shows that the winter temperature (December to February) is directly related to tree growth in the case of the *Abies spectabilis* collected near Dokriani glacier and the *Pinus wallichiana* collected near the Gangotri Glacier in UK (Supplementary Figure 4a,b). This relationship indicates that the Himalayan fir and pine trees conduct a significant amount of photosynthesis during warm winters and that the resultant photosynthates are used as stored food in the ensuing growing season. A similar relationship was observed for *Pinus wallichiana* in previous studies in the Himalaya^85, 86^. The analysis also showed positive correlations with the precipitation in May in the case of both the *Abies spectabilis* and *Pinus wallichiana* sampled from near the Dokriani and the Gangotri glaciers, respectively. This relationship was found to be statistically significant only in the case of samples collected near the Dokriani glacier. A rather significant negative growth relationship with the temperature of October of the previous year was observed for the samples collected near the Gangotri glacier. At first look, the correlation analysis of the samples taken near the Dokriani glacier and the Gangotri glacier suggests that, in this region and for the sampled tree species, the temperature plays a more significant role in determining tree growth than the precipitation. However, another fact is that the precipitation data from the extremely limited number of meteorological stations in the Himalayan region, which are ultimately used to generate the gridded datasets, are often not representational of the tree-ring sampling sites, as the precipitation in these mountains is mostly controlled by orography and shows significant variations between neighboring valleys^1^.

Samples of *Abies pindrow* were collected near the Solang valley in HP and the Thazwas valley in J&K. The growth of *Abies pindrow* in HP shows a statistically significant negative correlation with the temperature of the preceding October, November and July. In addition, it shows a statistically significant positive correlation with the precipitation in the month of February. The tree growth of *Abies pindrow* in J&K shows a strong negatively correlation with the summer temperatures (May-September). Subsequently, it shows a significant positive correlation with the total precipitation during the months of May and June. The analysis suggests that the increased precipitation and reduced temperature during the summer months in these arid (HP) and WD dominated (J&K) mountains favor growth by reducing the water losses caused by evapotranspiration.

### Mass balance reconstruction

The significant negative correlation between the tree-ring data and the Annual Specific Mass Balance (*MB*_*s*_) suggests that the tree-ring width of these alpine silver fir and pine could be a useful indicator of the *MB*_*s*_ variation. Correlations between the mass balance and tree-ring width index (RWI) chronologies for J&K, HP, and UK are −0.80 (p < 0.001), −0.81 (p < 0.001), −0.84 (p < 0.001), respectively. Based on these correlations, the following linear regression models (given as equations 1, 2, and 3) between the RWI and the *MB*_*s*_ were developed for each site (Supplementary Figure 5).

1 $$M{B}_{s}(UK)=2.85-3.31\,X\,RWI$$

2 $$M{B}_{s}(J\,{\&}\,K)=1.54-1.87\,X\,RWI$$

3 $$M{B}_{s}(HP)=3.72-4.16\,X\,RWI$$

The suitability of the tree-ring chronologies was assessed by using the threshold value of EPS 0.85^87^. The durations of 1931–2015, 1872–2013, 1861–2014, and 1903–2015 were used as the common period (Supplementary Table 1) for *Abies pindrow* (Thazwas Glacier), *Abies pindrow* (Solang Valley), *Abies spectablis* (Dokirini valley), and *Pinus wallichiana* (Gangotri valley), respectively for the statistical analysis of the chronology (Supplementary Fig. 3). Further, to ensure the reliability and stability of the regression model (Supplementary Fig. 5), various statistical tests are carried out and the associated errors are estimated (Supplementary Table 3) (Supplementary Fig. 6).

The coefficient of determination (R^2^) is interpreted as the proportion of the variability of the dependent variable explained by the model. An R^2^ that is closer to 1 is better for the model^88, 89^. The adjusted R^2^ is a correction to the R^2^ which takes into account the number of variables used in the model. It could be negative if the R^2^ is near zero. The variance explained by the regression model (Supplementary Fig. 5) accounted for 70.4%, 64% and 66% of the UK, JK and HP time-series, respectively. For the validation of the output of the regression model, various statistical tests have been used (Supplementary Table 3). The correlation coefficients for UK (−0.84), HP (−0.81), and J&K (−0.80) were found to be significant at the 99% confidence level. The root mean square error (RMSE) has been widely used as a validation parameter for the reliability of the tree-ring chronologies-based reconstructions^90^. The predicted residual error sum of squares (PRESS) RMSE is a form of cross-validation used to provide a summary measure of the fit of a model^91, 92^. In the present study, the RMSE ranged from 0.15 m w.e. to 0.85 m w.e., whereas the PRESS RMSE ranged from 0.17 m w.e. to 0.33 m w.e., thus indicating considerably small errors in the reconstructed values. The F-test was used to estimate the significance of the regression model^93^ and it also provided acceptable values for UK (47.75, p < 0.001), HP (48.14, p < 0.001), and J&K (42.24, p < 0.001). These values validate the statistical significance of the models. The Durbin-Watson statistical coefficient (DW) is the order one autocorrelation coefficient and is used to check that the residuals of the model are not autocorrelated^94^. The DW values were found to be between 1.44 and 2.49 for all models, indicating that the residuals of the model are not autocorrelated. The positive values of reduction of error (RE), and coefficient of efficiency (CE)^88, 89^ show significant skills in the reconstruction and acceptable model performances^90^.

### Cyclic behavior and spatial correlation of the reconstructed mass balance data

Wavelet analysis is a commonly used method for analyzing local variations of power within a time-series. The method involves the decomposition of a time-series into time-frequency space, which enables the determination of both the dominant modes of variability and their temporal variations^38^. In the present study, we used wavelet spectral analysis to determine the time-frequency domain of the annual specific mass balance (AS-MB) using the Interactive Wavelet Plot tool (http://ion.exelisvis.com/). The wavelet transforms demonstrated dominant periodic signals that could be correlated with the prevailing weather events (WD, ENSO, AMO) in the Himalaya. These signals vary in amplitude in a time-series and give information about changes in frequency. We also correlated the reconstructed annual specific mass-balance with the NOAA annual snow cover data interpolated for the Himalayan region (http://www.cpc.noaa.gov/data/snow/, Supplementary Figure 7) for nearly four decades (1972–2009). Theoretically, more snow cover should be associated with a more positive mass balance. The analysis shows positive correlations between the reconstructed mass balances and the snow cover for the entire Himalayan region. The correlation is most significant for the Indian Summer Monsoon (ISM) dominated regions (r > 0.6) and lower but still significant in the WD dominated regions (r > 0.3).

### Reliability of the reconstructed mass balance data

The published records of the field-estimated mass balance data for the Himalayan glaciers are very scarce and fragmented. Therefore, the reconstructed mass balance was compared with that of the mass balance estimated at regional scales using geodetic methods^95–98^ and degree-day method^99, 100^ (Supplementary Figure 8). In general, the mass balance reconstructed in the present study was found to be in agreement with the estimated regional geodetic mass balance and well within their error limits. Exceptions include a few cases involving the mass balance of only a single basin^100^ or a specific glacier^99^. It is interesting to note that the estimated mass balance of the Chhota Shigri glacier during 1986–2000^99^ is significantly less than the reconstructed mass balance, while the estimated mass balances during 1969–1985 and 2001–2012 for the same glacier^99^ show excellent agreement with our region-wide reconstructed mass balance. Thus, the reconstructed mass balance is in better agreement with the regional or long-term mass balance observations than with the glacier-specific or short-term mass balance observations. Further, the validation by comparison with geochronological and moraines dates through absolute dating techniques, i.e., radiocarbon (14 C) and terrestrial cosmogenic nuclide (TCN), as compiled by Rowan^47^ is not preferable here. For example, the TCN ages of moraines are single ages or more commonly the average of several samples and due to the large temporal uncertainty going up to ~200 years, it is not wise to directly correlate such geochronological data with our yearly reconstructed mass balances. Several studies^101–104^ have reported the glacier advances based on tree-ring dating in Tibet during the similar time frame as our study (1750 ± 20 CE). The estimated mass balances of the Karakoram region^96, 97^ display the famous ‘Karakoram anomaly’^105^ and are positive and significantly different from the reconstructed mass balance of the both HP and J&K glaciers, and therefore have not been included for this comparison.

### Data availability

The supporting datasets used for analyses during the current study are available as published literature and we have cited the source for accessing the data at appropriate places. The reconstructed mass balance dataset generated during the current study is available from the corresponding author on reasonable request. All data generated or analysed during this study are included in this published article (and its Supplementary Information files).

### Ethical approval and informed consent

We confirm that this study does not involve any biological experiments, harm to any animal species, or anthropogenic data collection.

## Electronic supplementary material

Supplementary Information
