# Supplementary material for: Himalayan glaciers experienced significant mass loss during later phases of little ice age
Source: Sci Rep. 2017 Sep 4;7:10305. doi: 10.1038/s41598-017-09212-2 (PMC5583174; doi:10.1038/s41598-017-09212-2)
Supplement: Supplementary file 1 — Supplementary Information [file 41598_2017_9212_MOESM1_ESM.doc]

**Himalayan glaciers experienced significant mass loss during later phases of little ice age**

Mayank Shekhar^1*^, Anshuman Bhardwaj^2^, Shaktiman Singh^3,4^, Parminder S. Ranhotra^1^, Amalava Bhattacharyya^1^, Ashish K. Pal^1^, Ipsita Roy^1^, F. Javier Martín-Torres^2,5^, María-Paz Zorzano^2,6^

^1^Birbal Sahni Institute of Palaeosciences, Lucknow, India

^2^Division of Space Technology, Department of Computer Science, Electrical and Space Engineering, Luleå University of Technology, Luleå, Sweden

^3^Institut für Kartographie, Technische Universität Dresden, Germany

^4^Department of Environmental Science, Sharda University, Greater Noida, India

^5^Instituto Andaluz de Ciencias de la Tierra (CSIC-UGR), Armilla, Granada, Spain

^6^Centro de Astrobiología (INTA-CSIC), 28850 Torrejón de Ardoz, Madrid, Spain

*Corresponding Author: Mayank Shekhar (email: [mayankshekhar01@gmail.com](mailto:mayankshekhar01@gmail.com))

**Supplementary figures**


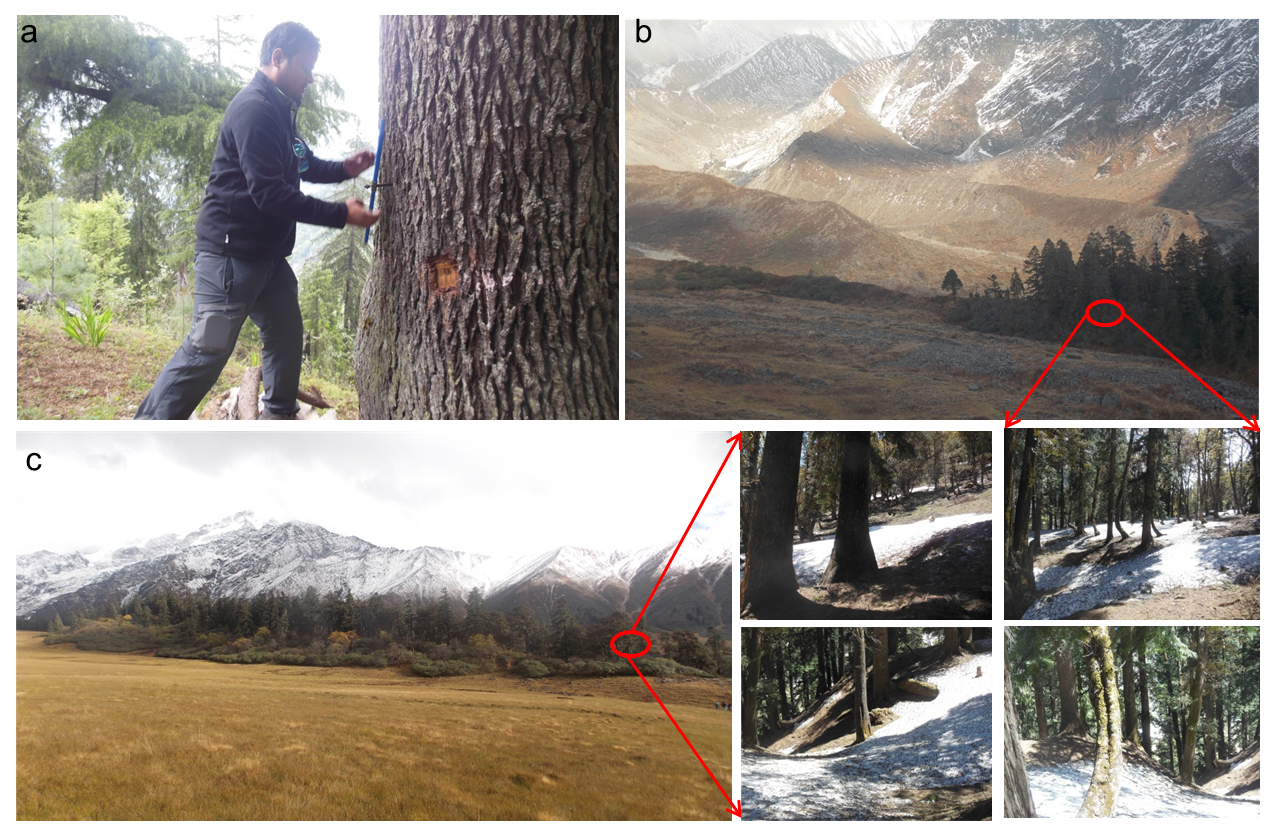


**Supplementary Figure |** **1. Field photographs from the Central and Western Himalayan regions. a,** Sample collection. **b, and c,** Sites of sampling in the uppermost tree line nearest to the glaciers. All the photographs used in this figure have been taken by the authors.

**
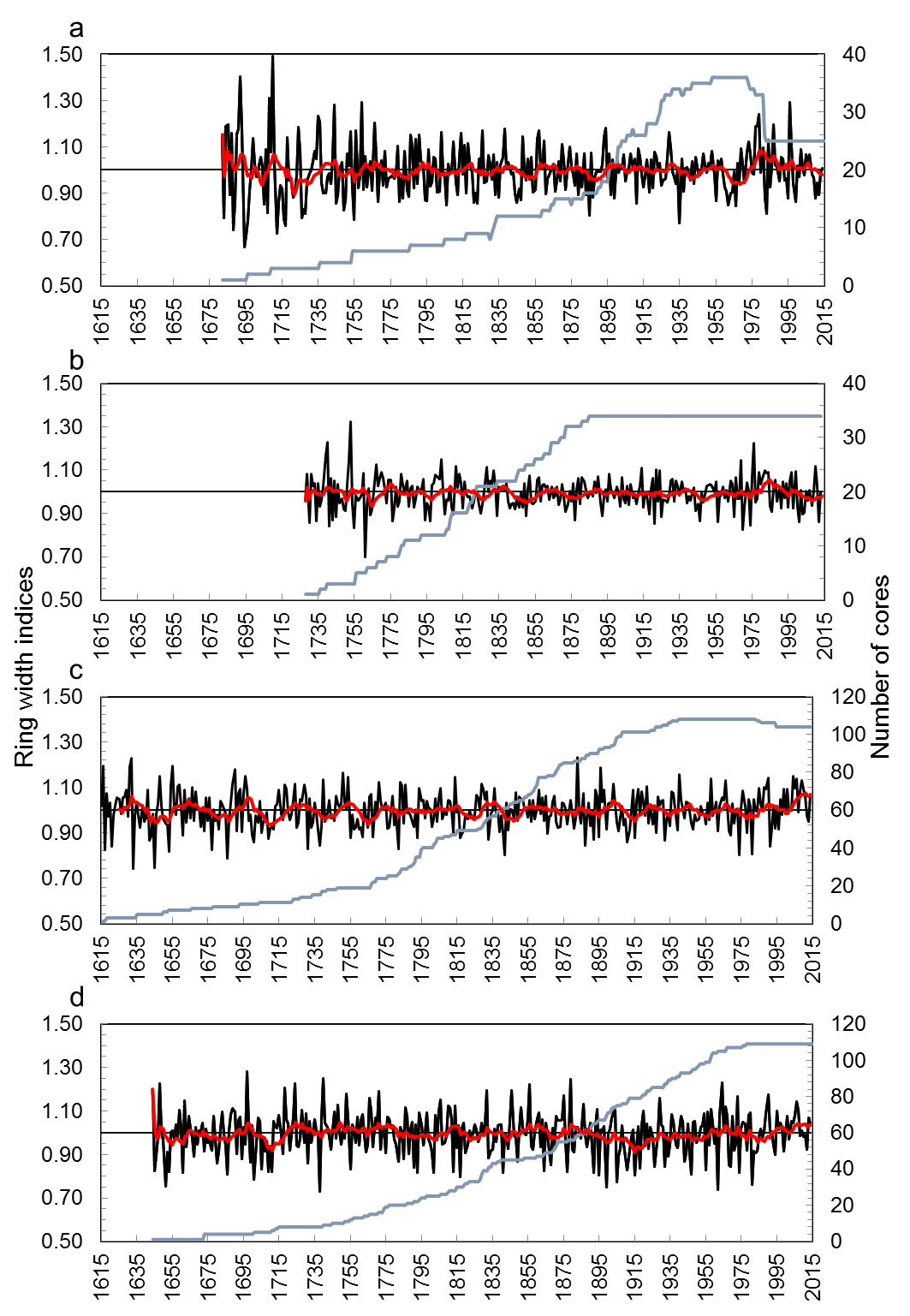
**

**Supplementary Figure |** **2.** **Plots of the residual tree-ring index chronologies with their sample depths.** **a,** *Abies pindrow* (Tajwah Glacier-J&K). **b,** *Abies pindrow* (Solang Valley-HP). **c,** *Abies spectablis* (Dokriani valley-UK). **d,** *Pinus wallichiana* (Gangotri valley-UK). The red line shows the eleven-year moving average of the ring width indices and the blue line shows the corresponding number of cores.


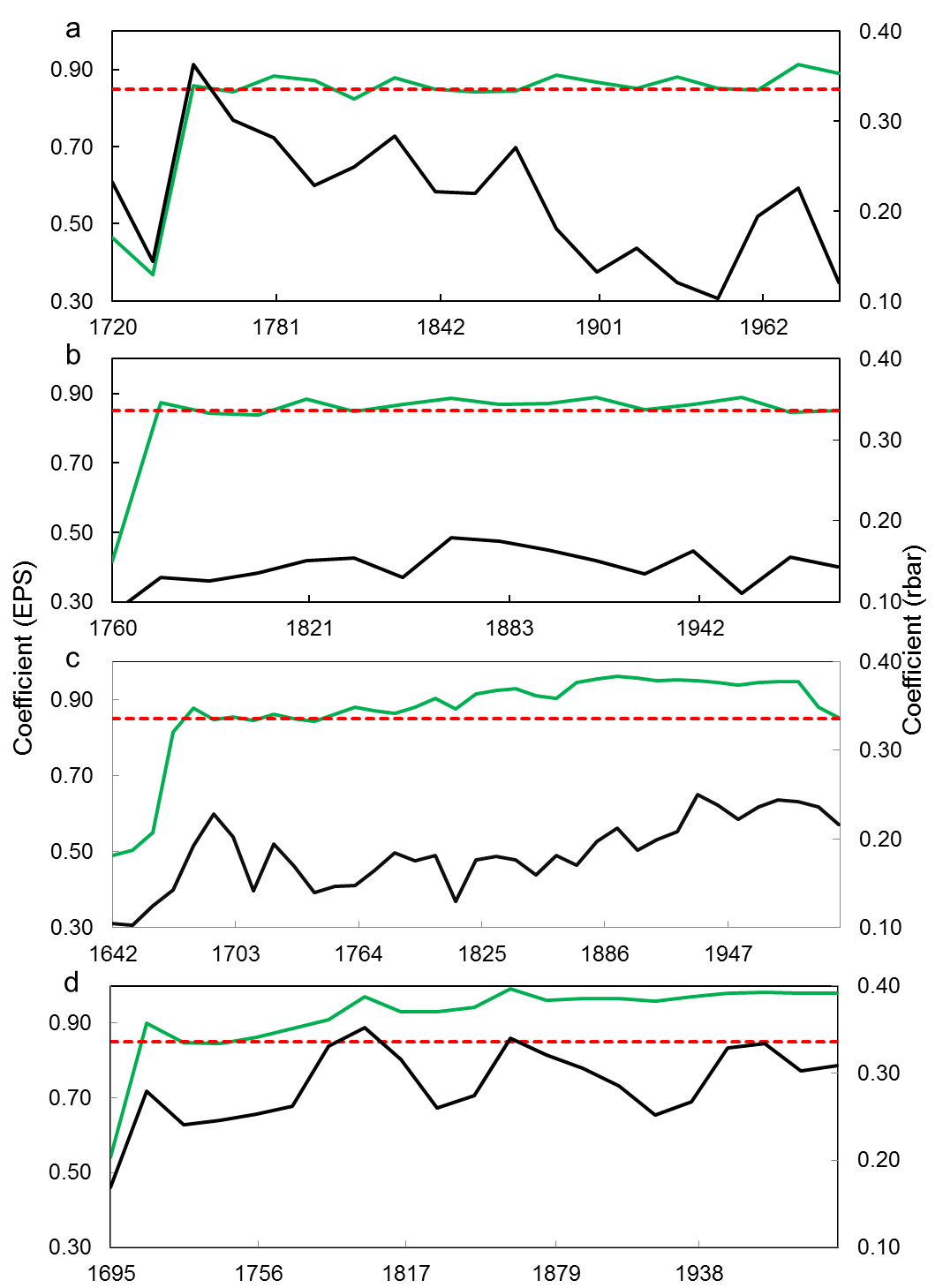


**Supplementary Figure | 3.** **Plots of running EPS and Rbar statistics. a,** *Abies pindrow* (Tajwah Glacier-J&K). **b,** *Abies pindrow* (Solang Valley-HP). **c,** *Abies spectablis* (Dokriani valley-UK). **d,** *Pinus wallichiana* (Gangotri valley-UK). The red dotted line shows the critical EPS threshold (0.85), values above which show the most significant reconstructions. The EPS (Green line) and Rbar (Black line) statistics are plotted with a 30-year window and a lag of 15 years.

**
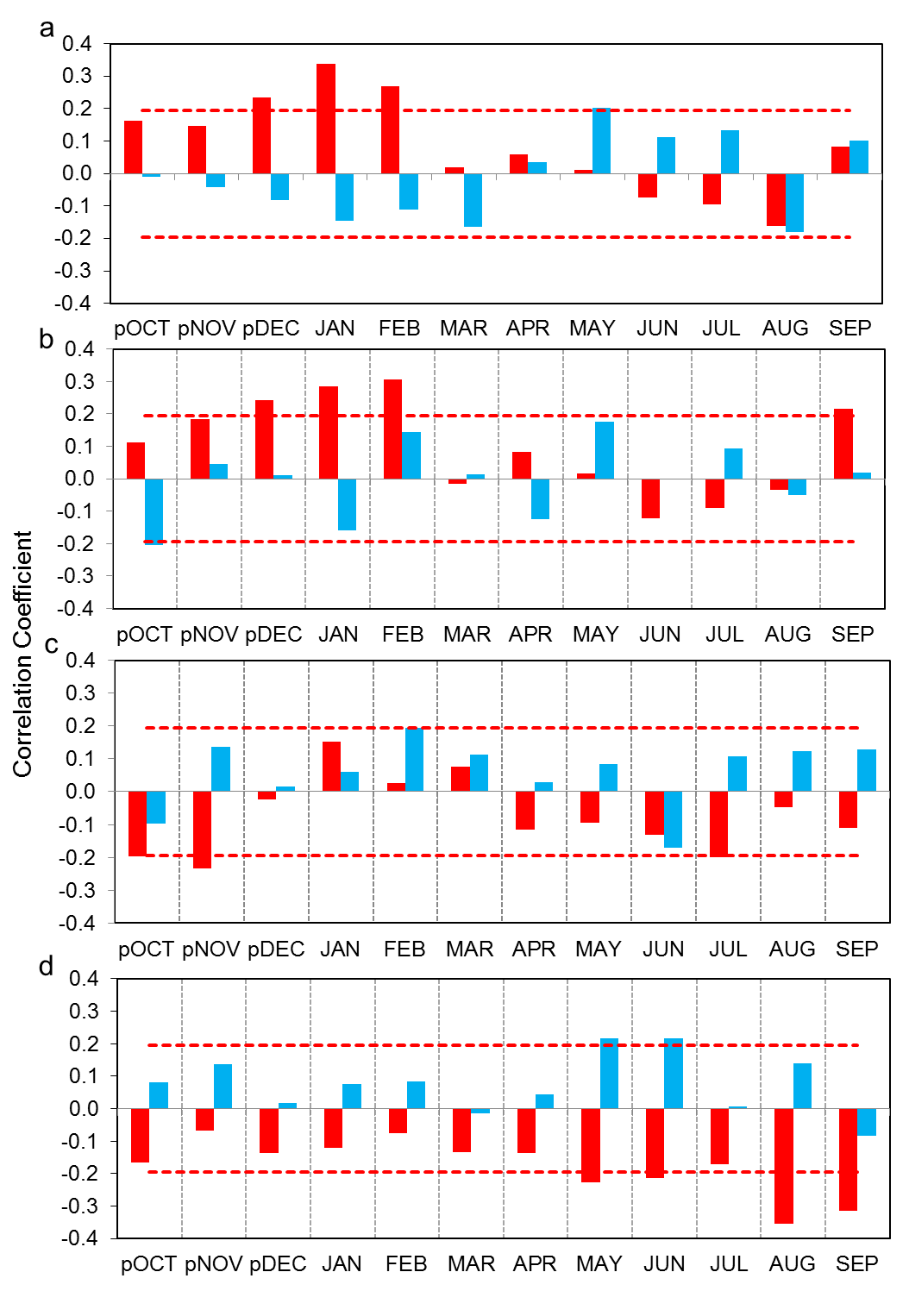
**

**Supplementary figure | 4. Correlation analysis of the residual chronologies with the mean monthly temperature (red bar) and total monthly precipitation (blue bar). a,** *Abies spectabilis* near the Dokirini glacier. **b,** *Pinus wallichiana* near the Gangotri Glacier. **c,** *Abies pindrow* near the Solang Valley. **d,** *Abies pindrow* near the Tajwah Glacier. The red dotted horizontal line indicates the 95% confidence level. Prefix ‘p’ before the months denotes the months of the previous growth year.


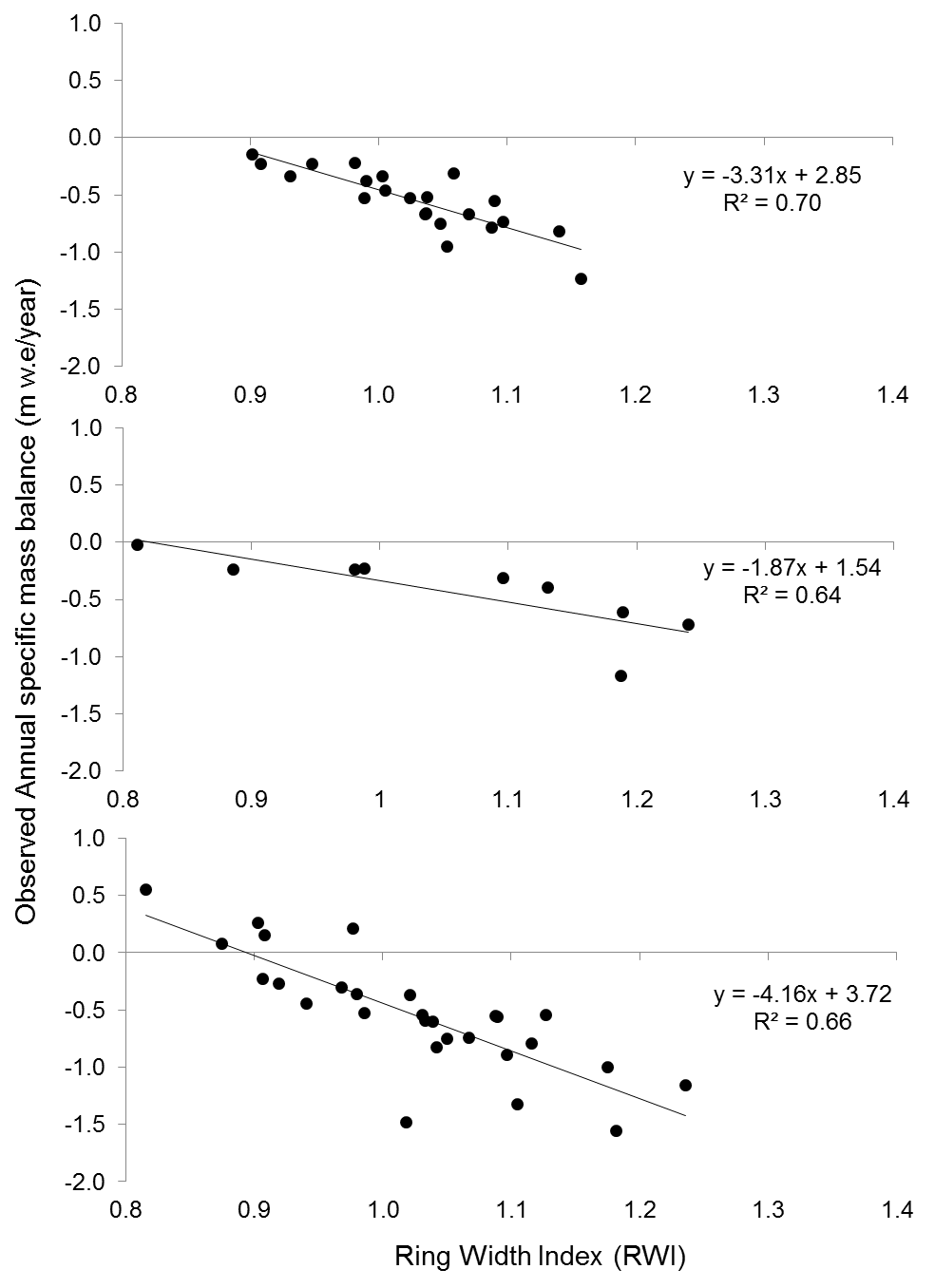


**Supplementary Figure | 5.** **Correlation between the ring width index and observed annual specific mass balance. a,** UK. **b,** J&K. **c,** HP.

**
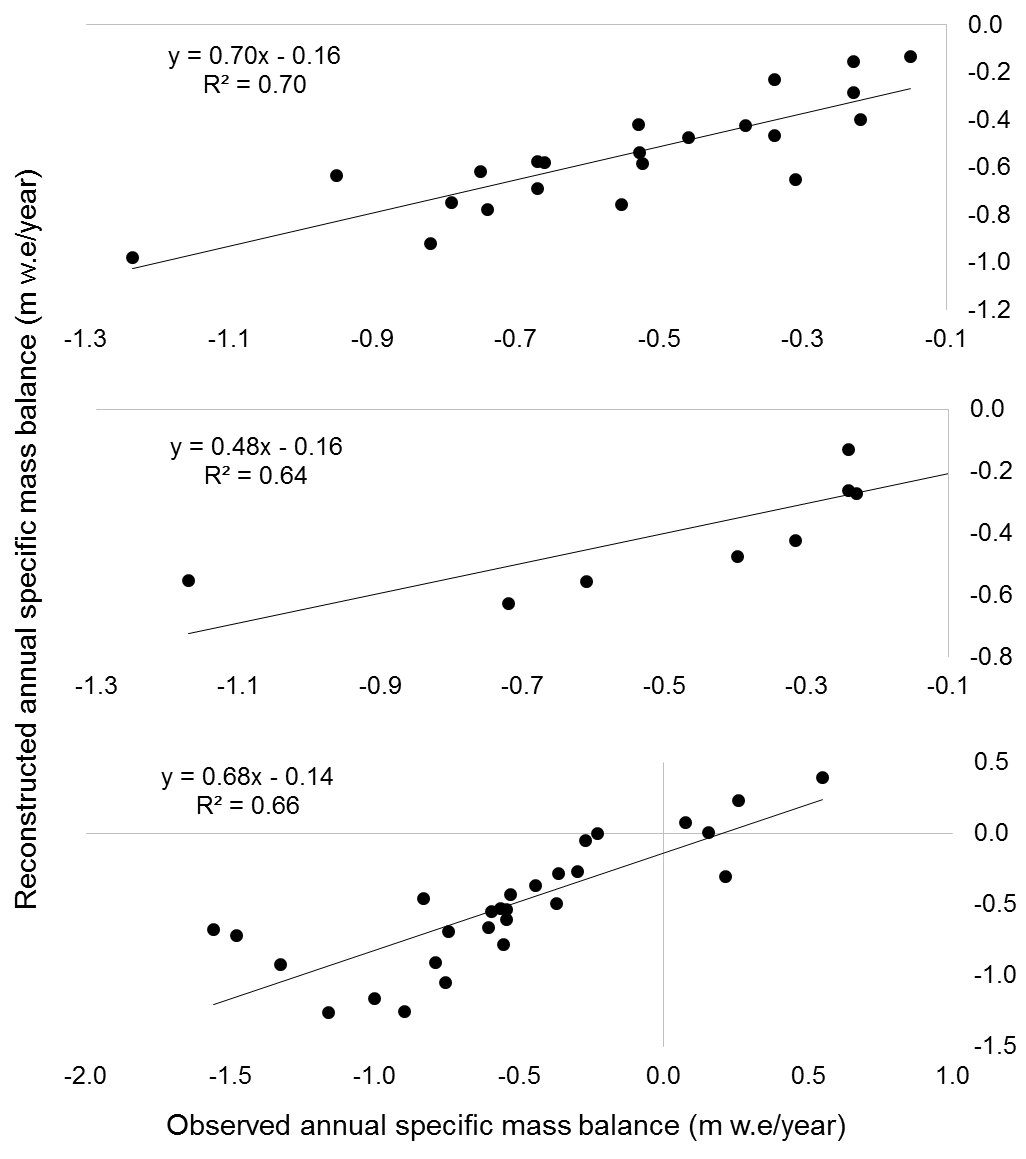
**

**Supplementary Figure | 6.** **Correlation between the observed annual specific mass balance and the reconstructed annual specific mass balance.** **a,** UK. **b,** J&K. **c,** HP.


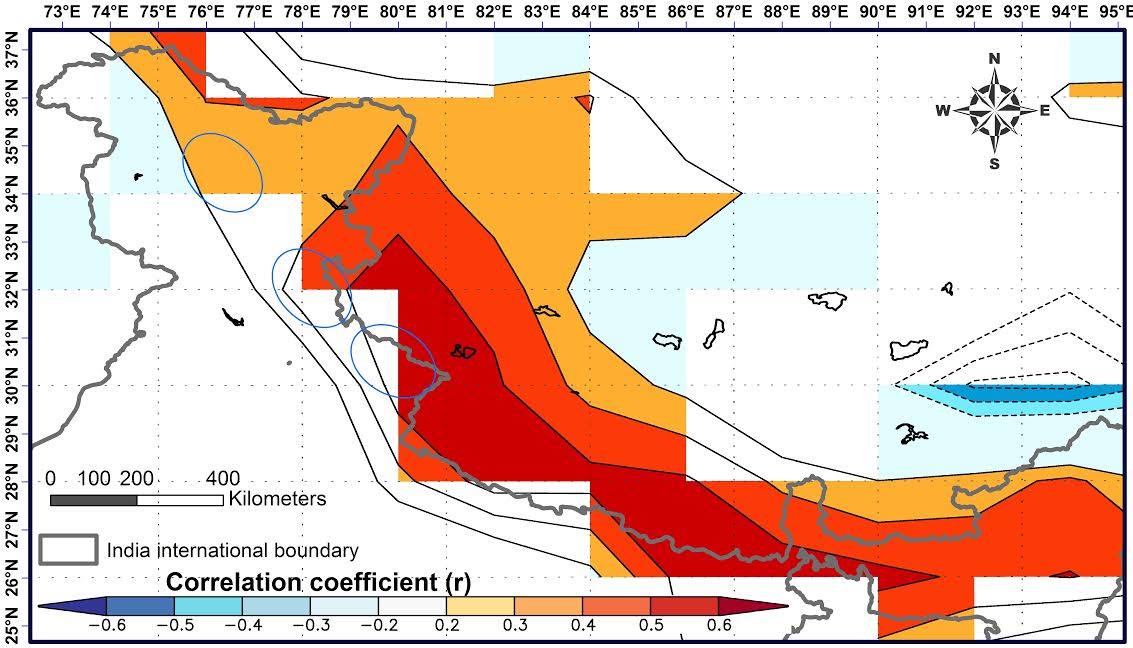


**Supplementary Figure | 7.** **Spatial correlation between the reconstructed AS-MB and NOAA snow cover data for the Himalayan region at an annual scale (1972-2009).** Blue ellipses show the representative reconstructed mass balance regions. The map is generated using ArcGIS Version 10.4 (http://desktop.arcgis.com/en/arcmap/latest/get-started/setup/arcgis-desktop-quick-start-guide.htm) and the KNMI Climate Explorer (http://www.knmi.nl)


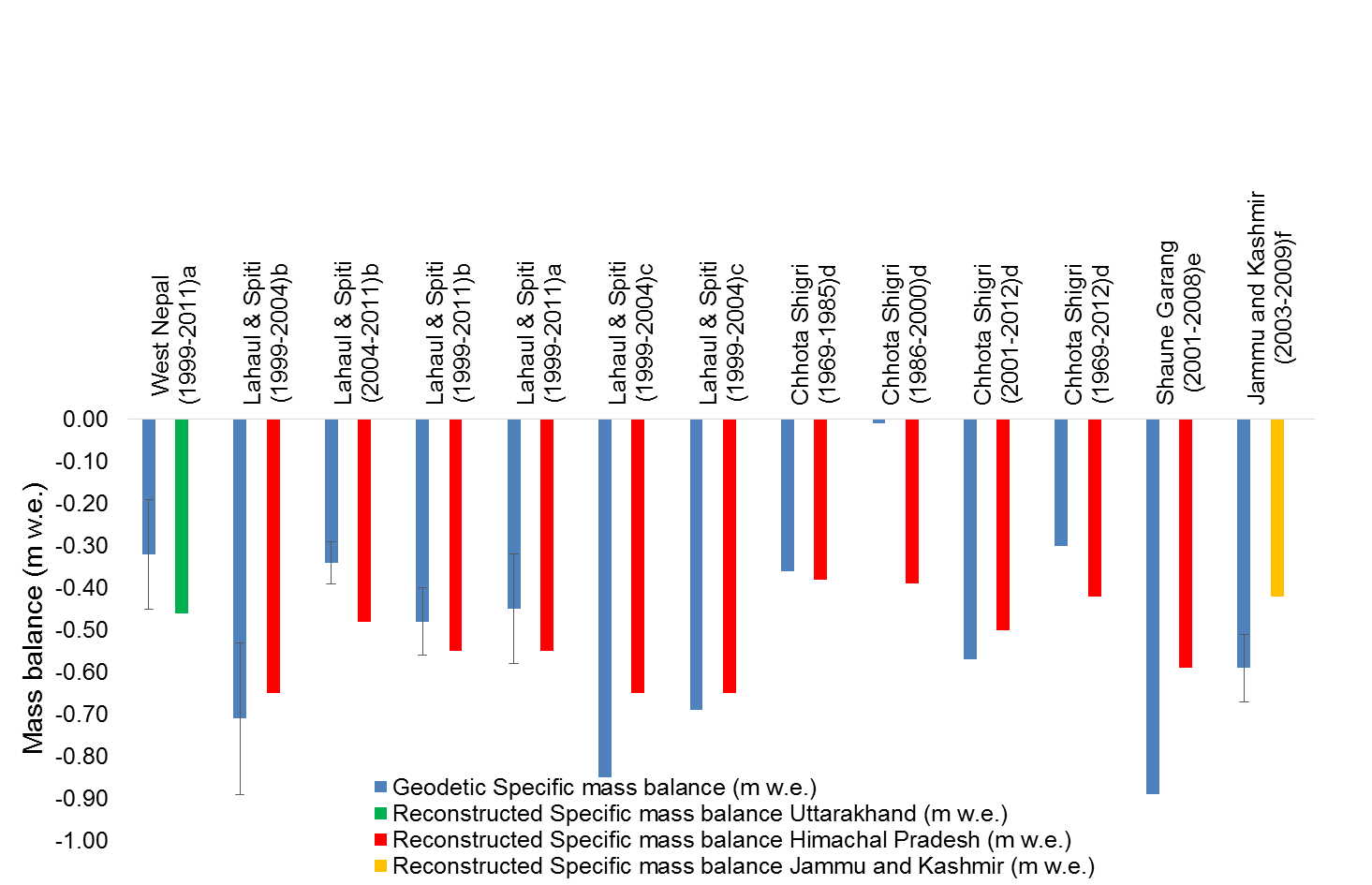


**Supplementary Figure | 8. The comparison of reconstructed mass balance with geodetic and degree-day mass balance estimates available for each region (a. Gardelle et al.^96^; b. Vincent et al.^98^; c. Berthier et al.^95^; d. Azam et al.^99^; e. Kumar et al.^100^; f. Kääb et al.^97^).**

**Supplementary tables**

**Supplementary Table | 1.** **Statistical characteristics of the chronologies.**

| **Statistical characteristics** | *Abies pindrow* (Tajwah Glacier) | *Abies pindrow* (Himachal Pradesh) | *Abies spectablis* (Dokriani valley) | *Pinus wallichiana* (Gangotri valley) |
| --- | --- | --- | --- | --- |
| **Chronology Time span** | 1682 -2015 | 1728-2013 | 1615-2014 | 1644-2015 |
| **Trees /Cores** | 14(27) | 28(35) | 78(112) | 83(122) |
| **Mean Sensitivity (MS)** | 0.26 | 0.29 | 0.18 | 0.17 |
| **Standard deviation (SD)** | 0.25 | 0.30 | 0.30 | 0.26 |
| **Common period** | 1931 – 2015 | 1872-2013 | 1861-2014 | 1903-2015 |
| **Mean correlation between trees (Rbt)** | 0.19 | 0.18 | 0.16 | 0.28 |
| **Mean correlation within-trees ( Rwt)** | 0.27 | 0.18 | 0.25 | 0.33 |
| **Mean correlation among all series (Rbar)** | 0.21 | 0.17 | 0.18 | 0.29 |
| **Signal to Noise ratio (SNR)** | 5.44 | 6.50 | 16.20 | 30.23 |
| **Expressed population signal (EPS)** | 0.85 | 0.87 | 0.94 | 0.97 |
| **~EPS Period > 0.85** | 1750- 2015 | 1775-2013 | 1670-2014 | 1710-2015 |

**Supplementary Table | 2. List of the glaciers with their observed mass balance data and the period of observation that was used for the mass balance reconstruction.**

| **Region** | **Name of glaciers** | **Area (sq. km)** | **Period of mass balance observation** |
| --- | --- | --- | --- |
| **Jammu and Kashmir** | Shishram | 9.91 | 1984 |
|  | Nehnar | 1.25 | 1976-1984 |
|  | Kolahoi | 11.91 | 1984 |
| **Himachal Pradesh** | Chhota Shigri | 15.7 | 1987-1989 and 2003-2011 |
|  | Gara | 5.2 | 1975-1983 |
|  | Gor Garang | 2.02 | 1977-1985 |
|  | Hamtah | 3.2 | 2001-2012 |
|  | Naradu | 4.56 | 2001-2003 |
|  | Shaune Garang | 4.94 | 1982-1991 |
| **Uttarakhand** | Chorabari | 6.6 | 2004-2010 |
|  | Dokriani | 7 | 1993-1995 and 1998-2000 |
|  | Dunagiri | 2.5 | 1985-1990 |
|  | Tipra Bamak | 7 | 1982-1989 |

**Supplementary Table | 3. Calibration and verification statistics for the regression models developed for the reconstruction of the** mass balance.

| **Period** | **Model** | **r** | **R^2^** | **R^2^ Adj.** | **F** | **RMSE (m w.e.)** | **PRESS RMSE (m w.e.)** | **RE** | **CE** | **DW** |
| --- | --- | --- | --- | --- | --- | --- | --- | --- | --- | --- |
| **1982-2010** | UK | -0.84 | 0.70 | 0.69 | 47.75 | 0.15 | 0.17 | 0.43 | 0.39 | 2.18 |
| **1976-1984** | J&K | -0.80 | 0.64 | 0.59 | 42.24 | 0.23 | 0.31 | 0.81 | 0.64 | 2.49 |
| **1975-2011** | HP | -0.81 | 0.66 | 0.64 | 48.14 | 0.31 | 0.33 | 0.52 | 0.45 | 1.44 |
